# An empirical analysis of the factors driving customers’ purchase intention of green smart home products

**DOI:** 10.3389/fpsyg.2023.1272889

**Published:** 2023-10-26

**Authors:** Mingyan Guo, Shufeng (Simon) Xiao

**Affiliations:** ^1^College of Business, Gachon University, Seongnam, Republic of Korea; ^2^Division of Business Administration, Sookmyung Women's University, Seoul, Republic of Korea

**Keywords:** green smart home products, sense of belongings, self-actualization, task-technology fit, social-technology fit, purchase intention

## Abstract

With the improvement of consumers’ environmental awareness and the popularity of the Internet of Things, green smart home products (GSHPs) are becoming the dominant trend of future home life. This shift not only makes tedious home life easier and more convenient but also helps families save energy and reduce carbon emissions. However, given the impact of the current technological level, the proportion of users who actually purchase GSHPs remains small. Thus, seeking ways to promote the consumption of GSHPs has become an urgent issue. Hence, this study seeks to fill the gap in the existing research on green consumption behavior and obtain a full understanding of the factors influencing the purchase intention of GSHPs. To do so, this work uses task-technology fit theory and considers the actual situation of green smart home consumption to add social-technology fit into the original theoretical basis. In particular, this research focuses on middle- and high-end Chinese consumers who have experience in purchasing GSHPs. Moreover, it aims for an in-depth exploration of the formation mechanism of Chinese consumers’ purchase intention for GSHPs through structural equation modeling. Using survey data collected from 331 green smart home product users in China, the study empirically examines the relationships among autonomy, environmental agility, sense of belonging, and self-actualization, and both task-technology fit and social-technology fit, which are expected to shape the purchase intention of GSHP users. The empirical results provide broad support for our hypotheses. The results of this study offer important contributions to the increasing research on GSHPs consumption and shed light on the importance of both technology characteristics and the needs of users in achieving both task-technology fit and social-technology fit and, ultimately enhancing the users’ intention to purchase GSHPs.

## 1. Introduction

With the rapid development of Internet technology and the continuous improvement of people’s demands for living environments, the shortcomings of traditional home products in terms of safety, resource waste, and lack of passivity have become increasingly prominent (Teoh et al., 2022). This circumstance has given rise to green smart home products (GSHPs). After installing a green smart home system, consumers can monitor their security system remotely. Furthermore, intelligent devices, such as smart door locks and high-definition cameras, can provide real-time protection for home safety throughout the day. These technologies are also environmentally friendly because smart homes are activated only when needed or when sensing an order. Thus they effectively avoid the wastage of water and electricity that often results from forgetting to turn off lights or close faucets. In terms of convenience, GSHPs can be connected into a single ecosystem, thereby enabling interoperability and intelligent control. Compatible with the modern and fast-paced life and work rhythm, GSHPs present a substantial advantage in liberating people from tedious chores, satisfying the demand for consumption upgrades, and ensuring a leisurely and relaxed life. Moreover, the thriving of smart homes will stimulate the development of the home products industry and related upstream and downstream industries, which can boost economic growth. However, given the impact of the current level of technology, the actual proportion of GSHP users remains small. As such, accelerating the potential of green smart home consumption has become an urgent problem. Utilizing the characteristics of GSHPs to accurately match consumers’ mental life needs has become a new research perspective.

In the extant literature, two different opinions have emerged regarding how consumers perceive GSHPs. On the one hand, some scholars have explored the positive effects of smart home technology on consumers’ purchase intentions. Smart homes, through centralized and intelligent control of lighting, heating, air conditioning, and other household systems and related devices, can provide consumers with optimal convenience, comfort, and safety while improving energy efficiency (Luor et al., 2015). Green smart home technology can simultaneously meet consumers’ functional and hedonic goals; moreover, using GSHPs allows consumers to save energy, control the ambient environment, enhance security, and provide additional entertainment and enjoyment (Wilson et al., 2017). Smart home technology enables consumers to access, manage, and monitor home products remotely via user interfaces on mobile devices, thus eliminating time and space constraints; it also allows consumers to control home devices remotely and contributes to the enhancement of the consumption experience (Yang et al., 2017). As innovative technological products, GSHPs can provide consumers with unprecedented techno-coolness, thus allowing them to experience technological advances and making their homes modern and futuristic. Techno-coolness can help consumers achieve complex psychological goals, such as enhancing interaction with others and self-achievement, thereby promoting consumers’ purchase intentions (Mamonov and Koufaris, 2020). GSHPs can aid consumers in achieving practical functionality and psychological goals. Meanwhile, these products can also reduce individuals’ environmental impact, particularly by controlling the use of energy and water in homes; in doing so, these technologies help consumers achieve their environmental goals through technological innovation (Schill et al., 2019; Sovacool and Del Rio, 2020).

On the other hand, some experts have raised concerns about the promotion of GSHPs. Home is an independent and private space where consumers seek shelter and sanctuary. The introduction of GSHPs may undermine consumers’ control of their home environment and trigger concerns about privacy loss (Hong et al., 2020; Mamonov and Koufaris, 2020). In the event of hacker attacks and technical failures, GSHP consumers are more likely to suffer the most losses (Yang et al., 2017). Although GSHPs can achieve energy conservation through technological means, they also consume a substantial amount of energy. Some energy-consuming features include automated security monitors and Internet connectivity. Backend services for these products, such as cloud storage servers, can also put pressure on energy resources. These challenges may have negative impacts on the consumption of GSHPs (Wilson et al., 2017). The adoption of GSHPs is a disruption to the existing home living habits and requires the continuous learning and adaptation of all family members. The learning and mastery of technology can be complicated and time-consuming, which may potentially result in an inadequate grasp of all the operations of GSHPs and the partial use of some functions. Moreover, the energy-saving potential claimed by GSHPs has not been confirmed in consumers’ actual use, which can also affect their consumption experience of these products (Hargreaves et al., 2018). Currently, consumers are not adequately familiar with green smart home technology and cannot fully adapt to the lifestyle changes brought about by these new products, thus potentially hindering the further promotion of GSHPs (Hong et al., 2020).

The existing literature on factors influencing consumers’ purchase intention for GSHPs has been inconsistent, and the relationship between smart home technology and consumers’ purchase intention remains unclear. Therefore, to fill the gaps in the existing research on green consumption behavior, this study aims to clarify the influencing factors of consumers’ purchase intentions for GSHPs by empirically analyzing how the characteristics of GSHPs, consumers’ needs, and their fitness work in the process of consumption decision-making. Inspired by task-technology fit theory and considering the actual situation of green smart home consumption, this study adds social-technology fit to the original task-technology fit framework. Furthermore, this study focuses on Chinese middle- and high-end consumers who have purchased GSHPs, the study aims to explore the formation mechanism of Chinese consumers’ purchase intention for GSHPs. In this research, autonomy and environmental agility are considered as the technology characteristics of GSHPs, while consumers’ needs for a sense of belonging and self-actualization are regarded as task features. The technology characteristics and task features are studied as independent variables. Moreover, task-technology fit and social-technology fit act as mediating variables, and purchase intention for GSHPs is the dependent variable. This research aims to understand the factors affecting consumers’ purchase intention for GSHPs comprehensively. It seeks to broaden the scope of existing green consumption research and contribute to the popularization of GSHPs and the maximization of their consumption potential.

## 2. Theoretical background and hypothesis development

Abraham Maslow’s hierarchy of needs theory aims to explain the relationship between human needs and behavior, which asserts that human needs will influence their behavior. Maslow has divided human needs into five levels, and from the lowest to the highest are physiological needs, safety needs, belongingness needs, esteem needs, and self-actualization needs, respectively. Each level is interconnected and built on the level below it; one can only progress to higher-level needs when lower-level needs are met (Maslow, 1987; Fives and Mills, 2016). In the research field of consumer behavior, the hierarchy of needs is widely applied to studies related to consumers’ purchase intentions and behavior, such as motivations for purchasing electric vehicles (Cui et al., 2021), adoption of virtual reality technologies for ocean conservation (Yuen et al., 2022), and usage preferences for reusable delivery bags (Su et al., 2023).

However, as technology advances rapidly, focusing the research solely on needs cannot fully explain consumers’ purchase behavior. Determining whether technology can meet consumers’ needs and the extent to which it can satisfy different needs is equally important. Therefore, this study also refers to task-technology fit theory. Task-technology fit involves the relationship between a specific technology and individual performance, which includes technology characteristics, task characteristics, and task-technology fit as the three main structures. Technology characteristics are the key features of a product that allow users to complete related tasks. Meanwhile, task characteristics refer to the outcomes users expect to achieve by using technology (Goodhue, 1995; Zhou et al., 2010). Technology characteristics and task characteristics are the antecedent variables affecting task-technology fit, which entails that users’ adoption of a particular technology depends on the match between technology and their task requirements. Users will adopt the technology only if it can match their tasks and improve their performance (Goodhue, 1995; Goodhue and Thompson, 1995). Task-technology fit theory has been applied to users’ technology adoption behaviors, such as mobile banking (Zhou et al., 2010; Oliveira et al., 2014), MOOCs (Wu and Chen, 2017), purchase intention of organic food (You et al., 2020), augmented reality technology (Faqih and Jaradat, 2021), and smart speakers (Ling et al., 2021).

**Figure 1:**
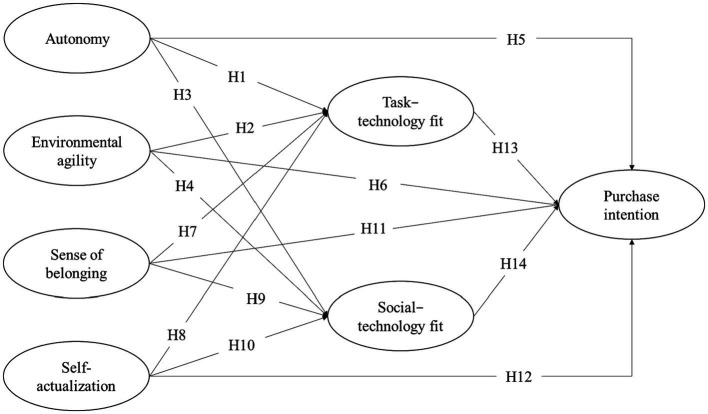
Research model and proposed hypotheses.

This study aims to fill the research gap in consumers’ green purchase behavior by exploring the relationship between the characteristics of GSHPs and the needs of contemporary consumers. In addition, it clarifies the factors influencing consumers’ GSHP purchase intention. This study refers to task-technology fit theory and uses the autonomy and environmental agility of GSHPs as the technology characteristics. With the guidance of the hierarchy of needs theory, it considers the sense of belonging and self-actualization as the task characteristics. We present the conceptual framework in Figure 1. As shown in Figure 1, the theoretical model is constructed with technology characteristics and task characteristics as independent variables, task-technology fit and social-technology fit as mediating variables, and consumers’ GSHPs purchase intention as the dependent variable.

### 2.1. Technology characteristics and task-technology fit

Task-technology fit is the core element of the task-technology fit model, which refers to the degree of fit between the characteristics of technology and the tasks that the users need to accomplish (Goodhue, 1995; Yang et al., 2022). One of the technology characteristics of GSHPs is autonomy, which means that they can operate independently in a goal-directed way (Rijsdijk et al., 2007; Rokonuzzaman et al., 2022). Guided by orders from a computer, smartphone, or intelligent speaker, GSHPs can perform the expected operations automatically (Yang et al., 2017). Given its autonomy, GSHPs can reduce time and space constraints during utilization compared with traditional products (Hoffman and Novak, 2018). An increasing number of smart home devices, including electrical appliances, lighting, and security devices, have transformed homes into fully automated residences (Aldrich, 2003; Lee, 2020). In a smart home, consumers can activate their home mode through a smart system app, voice command, or even a gesture. When users return home, the connected smart products can respond automatically and simultaneously, such as by turning on the lights, playing background audio and television, closing the curtains, and activating security systems. The functions of a smart home product can be customized according to individual needs; moreover, the devices can be controlled automatically on the basis of the settings or requirements of the users for an optimal experience (Cook, 2012). Autonomy facilitates the efficiency of home products, saves time, and makes home life more convenient (Luor et al., 2015). In summary, the autonomy of GSHPs will facilitate the satisfaction of consumers’ needs.

Another significant feature of GSHPs is environmental agility, which refers to the ability of GSHPs to observe the surrounding environment and respond to environmental changes and user requirements (Rokonuzzaman et al., 2022). GSHPs can not only react to their surroundings but also actively explore and analyze the environment through built-in sensors (Rijsdijk and Hultink, 2009; Hoffman and Novak, 2018). Relying on the observations, GSHPs can perform operations through built-in actuators and provide intelligent services to their users (Raff et al., 2020). Environmental agility allows home products to learn from and respond to external environments. For instance, smart air conditioners can adjust modes according to indoor temperature and humidity changes, and smart lighting systems can automatically turn on or off depending on the changes in ambient brightness (Cook, 2012). Environmental agility also has great potential to reduce household energy consumption, improve energy utilization, and achieve green environmental goals (Rokonuzzaman et al., 2022). In summary, the environmental agility of GSHPs will have a positive impact on task-technology fit. The above discussion leads to the following hypotheses:

*Hypothesis 1*: Autonomy has a positive impact on task-technology fit.

*Hypothesis 2*: Environmental agility has a positive impact on task-technology fit.

### 2.2. Technology characteristics and social-technology fit

Social-technology fit refers to the extent to which technology can help users fulfill their social needs (Lu and Yang, 2014). The autonomy of GSHPs emphasizes automation and independence in the operation of GSHPs (Rijsdijk et al., 2007; Rokonuzzaman et al., 2022). Environmental agility implies that GSHPs not only respond to external changes in the surrounding environment but also have the potential to optimize their responses by collecting and processing information from the surroundings (Rokonuzzaman et al., 2022). Besides the ability to manage internal household devices, predict users’ needs, and respond to these needs, smart home technology can also help its users establish connections with the outside world (Aldrich, 2003). Compared with traditional home products, GSHPs have a higher level of intelligence, thus giving consumers a sense of superiority (Rijsdijk and Hultink, 2009). Autonomy and environmental agility demonstrate the technological innovation of home products. The techno-coolness of smart thermostats helps consumers to project an improved self-image and satisfy their expectations for social recognition, reputation, and social status (Mamonov and Koufaris, 2020). Meanwhile, the global energy crisis and rising energy prices have raised consumers’ environmental awareness. Related research has found that the consumption of green products will be beneficial for establishing a better social status and improving personal image (Khan and Mohsin, 2017). The autonomy and environmental agility of GSHPs can reduce energy consumption in home life. Thus, the purchase of GSHPs is expected to contribute to establishing a user’s green consumer image. In summary, the autonomy and environmental agility of GSHPs are expected to have a positive impact on social-technology fit. The above discussion leads to the following hypotheses:

*Hypothesis 3*: Autonomy has a positive effect on social-technology fit.

*Hypothesis 4*: Environmental agility has a positive effect on social-technology fit.

### 2.3. Technology characteristics and consumers’ purchase intention

Technology characteristics provide performance warranties and sources to ensure consumer confidence. Moreover, users’ perception of the technology characteristics will indicate the potential adoption of smart home technology (Wilson et al., 2017). Earlier studies have found that the level and reliability of smart home technology is the most important deterministic factor influencing consumers’ acceptance of smart home products (Schomakers et al., 2021; Li et al., 2023). As the most distinctive features of smart home technology, the autonomy and environmental agility of GSHPs will directly influence consumers’ purchase intention for such products, along with a high perception of autonomy and environmental agility and a high likelihood of purchase intention. The above discussion leads to the following hypotheses:

*Hypothesis 5*: Autonomy positively affects consumers’ purchase intention for GSHPs.

*Hypothesis 6*: Environmental agility has a positive effect on consumers’ purchase intention for GSHPs.

### 2.4. Task characteristics and task-technology fit

Task-technology fit refers to the degree of fit between task characteristics and technology characteristics (Goodhue, 1995). According to Maslow’s hierarchy of needs, people are always seeking to satisfy higher-level needs. A sense of belonging arises when physiological and safety needs are largely satisfied; furthermore, people yearn for intimate relationships with others (Maslow, 1987; Fives and Mills, 2016). The need for belongingness emphasizes an individual’s emotional involvement with a certain group, thus implying the alignment of personal goals with those of other group members and the acquisition of social recognition from the group (Cheung and Lee, 2012). People always desire opportunities to connect and communicate with others. The adoption of health-related products and technologies by the elderly mainly depends on whether the technology and products can enhance their sense of belonging (Thielke et al., 2012). In other words, the stronger the consumers’ need for a sense of belonging is, the more likely they will expect innovative technology to meet their needs. Therefore, a sense of belonging is expected to have a positive impact on task-technology fit.

Self-actualization is the highest level of need in Maslow’s theory. When other needs are met, people will seek to satisfy their need for self-actualization through participating in creative activities (Thielke et al., 2012). Self-actualization is a fundamental motivation for self-development and self-improvement and is closely related to an individual’s sense of self-fulfillment. It refers to the desire to achieve one’s unique and idiosyncratic existence (Taormina and Gao, 2013). Furthermore, a strong need for self-actualization will result in a high willingness to engage in consumption behavior (Fraj and Martinez, 2006). The adoption of GSHPs can provide a platform for users to acquire knowledge and resources for self-improvement and personal growth (Zhao et al., 2011). Consumers with a stronger need for self-actualization will show greater expectations of GSHPs for satisfying their needs. Therefore, self-actualization is expected to have a positive impact on task-technology fit. The above discussion leads to the following hypotheses:

*Hypothesis 7*: Sense of belonging has a positive effect on task-technology fit.

*Hypothesis 8*: Self-actualization has a positive effect on task-technology fit.

### 2.5. Task characteristics and social-technology fit

Social-technology fit emphasizes the degree of fit between social needs and technology, where social needs encompass perceptions of image, social recognition, and others’ evaluations (Ashfaq et al., 2021). Sense of belonging emphasizes the attachment relationship of an individual with a certain group (Cheung and Lee, 2012; Hawkins, 2020), while self-actualization represents the pursuit of an individual for uniqueness and self-fulfillment (Taormina and Gao, 2013). When individuals have a stronger belief in their identity as a group member, they will perceive a greater sense of belonging. Thus, they will be more willing to define themselves by their group membership and gain a sense of recognition from their group (Hawkins, 2020). With consideration of users’ needs for belongingness, manufacturers of GSHPs have introduced functions that allow smart devices to connect with the devices of users’ relatives or friends; these functions enable interaction with others through sharing information or inter-operating systems and social connections that are unrestricted by physical space (Lee et al., 2017). As living spaces, houses are the reflection of residents’ lives and, to some extent, represent their identity and status. Compared with traditional home products, the use of GSHPs can indicate consumers’ pursuit of a higher quality of life, thus revealing their identity and social status (Gram-Hanssen and Darby, 2018). In summary, the greater consumers’ needs for a sense of belonging and self-actualization are, the stronger they hope to fulfill their social needs through smart home technology. The above discussion leads to the following hypotheses:

*Hypothesis 9*: Sense of belonging has a positive effect on social-technology fit.

*Hypothesis 10*: Self-actualization has a positive effect on social-technology fit.

### 2.6. Task characteristics and consumers’ purchase intention

Researchers have provided some empirical evidence for the relationship between a sense of belonging, self-actualization, and consumers’ purchase intention. Sense of belonging has a statistically direct influence on citizen participation in smart city projects (Lebrument et al., 2021). Meanwhile, the differences in the need degree for belonging result in discrepant purchase behaviors for counterfeit consumption (Hawkins, 2020). The pursuit of self-actualization leads to socially conscious consumption and has a direct positive association with consumers’ repurchase intention for fair-trade coffee (Hwang and Kim, 2018). Therefore, a sense of belonging and self-actualization may generate a direct influence on consumers’ purchase intention for GSHPs with their need for a better attachment to their groups and desire for personal growth. The above discussion leads to the following hypotheses:

*Hypothesis 11*: Sense of belonging has a positive effect on consumers’ purchase intention for GSHPs.

*Hypothesis 12*: Self-actualization has a positive effect on consumers’ purchase intention for GSHPs.

### 2.7. Task-technology fit and consumers’ purchase intention

Consumers’ purchase intention indicates that consumers are prepared to engage in purchase behavior; moreover, it is considered a direct antecedent toward purchase behavior (Lu and Yang, 2014). According to task-technology fit theory, the adoption of technology depends on the matching degree of its characteristics and the users’ task needs. When the task-technology fit is high, increasing the technology usage rate and improving users’ performance is possible (Goodhue, 1995; Goodhue and Thompson, 1995). Studies in various research domains have verified the relationship between task-technology fit and usage. For example, if the immediacy of mobile banking can satisfy consumers’ needs for mobile transactions, it can positively promote consumers’ adoption of mobile banking (Zhou et al., 2010). At the same time, if the information and entertainment function of a smart speaker can fit their user’s needs for information gathering and entertainment enjoyment, consumers’ purchase intention for smart speakers will be promoted by the good task-technology fit (Ling et al., 2021). In summary, task-technology fit will have a positive impact on consumers’ purchase intention for GSHPs. The above discussion leads to the following hypotheses:

*Hypothesis 13*: Task-technology fit has a positive effect on consumers’ purchase intention for GSHPs.

### 2.8. Social-technology fit and consumers’ purchase intention

Previous research has shown that a better social-technology fit will contribute to users’ intention to adopt online social network sites (Lu and Yang, 2014). At the same time, if the technology of a certain online social platform has a high degree of fit with users’ social needs, users will experience high participation satisfaction; meanwhile, the satisfaction for information and knowledge sharing will also be improved (Wu et al., 2015). In summary, if the degree to which smart home technology meets social needs is high, consumers’ purchase intention for GSHPs will be improved. The above discussion leads to the following hypotheses:

*Hypothesis 14*: Social-technology fit has a positive effect on consumers’ purchase intention for GSHPs.

## 3. Methodology

### 3.1. Sampling and data collection

According to a survey by Statista, China’s smart home market is developing rapidly with global smart accessory manufacturers and IoT-related companies such as Siemens and Schneider Electric jumping into the Chinese market. The revenue of the smart home market in China reached US$26.67 billion, thereby ranking second in the world after the United States. Between 2022 and 2027, China’s smart home market is expected to experience an annual growth rate of 14.36%. Furthermore, 164 million Chinese households will become active users of smart homes by 2027, with household penetration increasing from 16.6% in 2022 to 34% by 2027. Considering the scale, growth potential, and speed of China’s smart home market, this study determines that focusing on middle- and high-end green consumers in China as the research target is of high research value. To assure the accuracy of the research target selection, we first found through research that Chinese green smart home consumption is mainly concentrated in the economically developed first- and second-tier cities in China. After obtaining a list of green smart home enterprises and regional consumption information from the Ministry of Industry and Information Technology of China, we commissioned a well-known local survey company in China to conduct a random survey of GSHP users in Shanghai, Beijing, and Guangzhou. A total of 387 questionnaires were collected in this survey, of which 331 were valid, with an efficiency rate of 85.53%.

To test the possibility of potential nonresponse bias in our study, we compared the differences in key demographic variables (e.g., user age, education level, and monthly income) between early-responding and late-responding participants. The results of the *t*-tests indicated no statistically significant differences (*p* > 0.05) in these users, thus suggesting that nonresponse bias was less likely to be a serious problem in our data. Furthermore, following the procedure recommended by Podsakoff et al. (2003), we also checked for the possible presence of common method variance (CMV) in our data by performing Harman’s one-factor analysis. We performed exploratory factor analysis using the principal factors method by including all multiple-item scales in an unrotated factor structure. The results of the analysis indicated that no general factor was apparent in the unrotated factor structure and accounted for the majority (i.e., more than 50%) of the variance, thereby providing no evidence of potential CMV concern in the study.

### 3.2. Variables and measurement

Unless otherwise noted, we measured all main variables of interest by adopting multiple-item, seven-point Likert scales (1 = strongly disagree, 7 = strongly agree). All the measures used to assess these variables were well-developed ones in the literature. We summarized the variables and their detailed measurement in Table 1.

**Table 1:** Results of construct reliability and validity assessments.

Construct and indicators	Mean	STD	Outer loading	Alpha	CR	AVE
Autonomy (ATM; Rokonuzzaman et al., 2022)				0.911	0.944	0.849
ATM1: A green smart home product does not need a lot of human inputs to function.	5.184	1.366	0.924			
ATM2: A green smart home product works independently.	4.997	1.352	0.922			
ATM3: A green smart home product finds its own way.	4.918	1.428	0.917			
Environmental agility (EA; Rokonuzzaman et al., 2022)				0.881	0.926	0.807
EA1: A green smart home product scans its environment.	6.103	1.161	0.864			
EA2: A green smart home product reacts to changes in the environment.	5.752	1.282	0.919			
EA3: A green smart home product directly adapts its behavior to the environment.	5.867	1.164	0.910			
Sense of belonging (SOB; Cheung and Lee, 2012)				0.930	0.955	0.877
SOB1: If other GSHPs users planned something, I would think of as something “we” would do rather than something “they” would do.	5.015	1.544	0.939			
SOB2: I see myself as a part of the GSHPs system.	4.764	1.468	0.935			
SOB3: In general, GSHPs make me feel a sense of belonging.	4.843	1.547	0.935			
Self-actualization (SA; Phang et al., 2006)				0.925	0.952	0.869
SA1: Using GSHPs gives me an opportunity for personal growth.	4.864	1.561	0.927			
SA2: Using GSHPs increases my feeling of self-fulfillment.	4.804	1.395	0.938			
SA3: Using GSHPs gives me a feeling of accomplishment.	4.822	1.516	0.932			
Task and technology fit (TTF; Lin and Huang, 2008; Zhou et al., 2010)				0.913	0.945	0.851
TTF1: The functionalities of GSHPs were very adequate.	5.450	1.344	0.919			
TTF2: The functionalities of GSHPs were very sufficient.	5.178	1.465	0.919			
TTF3: In general, the functionalities of GSHPs were best fit the task.	5.520	1.301	0.930			
Social and technology fit (STF; Lu and Yang, 2014)				0.911	0.944	0.849
STF1: In my opinions, GSHPs’ functions are suitable for helping me complete my social situation.	5.205	1.460	0.920			
STF2: In my opinions, GSHPs are enough to help me complete my social situation.	4.921	1.403	0.931			
STF3: In my opinions, GSHPs are fit for the needs of my social situation.	4.734	1.668	0.913			
Purchase intention (PIN; Cui et al., 2021)				0.849	0.909	0.769
PIN1: I often purchase GSHPs.	4.746	1.677	0.836			
PIN2: I plan to buy GSHPs.	5.251	1.317	0.908			
PIN3: I will buy GSHPs in the future.	5.589	1.317	0.885			

*N* = 331. STD, Standard deviation; Alpha, Cronbach’s alpha; CR, Composite reliability; AVE, average variance extract.

## 4. Analyses and results

### 4.1. Measure reliability and validity assessment

We used the structural equation modeling (SEM) method to test our proposed conceptual model empirically. Before testing the proposed hypotheses, we examined the reliability and validity of the constructs used in the study by estimating a measurement model. Table 1 presents the results of reliability and validity assessments. As shown in Table 1, the outer factor loadings of all research constructs were statistically significant (*p* < 0.001) and higher than the commonly accepted benchmark of 0.70. Moreover, the Cronbach’s alpha and composite reliability (CR) values for each of the constructs were higher than 0.70. These results demonstrated the strong reliability of all the constructs in the model (Fornell and Larcker, 1981). In addition, we calculated the average variance extracted (AVE) values to assess the convergent validity of the constructs. Table 1 shows the AVE values for each of the constructs were higher than the recommended threshold of 0.50, suggesting an adequate convergent validity of the measures (Fornell and Larcker, 1981). Finally, we assessed the discriminant validity of the measures by comparing the root values of the AVEs for each of the corresponding construct and the correlation coefficients between the construct and all the others in the model. The results revealed that the square root values of the AVEs for each construct were higher than the correlation coefficient values between the construct and all other constructs, which exhibited a strong discriminant validity of the measures used in the study (Table 2).

**Table 2:** Correlations and discriminant validity among the constructs.

Variables	1	2	3	4	5	6	7
1. Autonomy	***0.921***						
2. Environmental agility	0.190	***0.898***					
3. Sense of belonging	0.290	0.307	***0.937***				
4. Self-actualization	0.486	0.319	0.628	***0.932***			
5. Task-technology fit	0.463	0.361	0.633	0.653	***0.923***		
6. Social-technology fit	0.420	0.291	0.708	0.663	0.635	***0.922***	
7. Purchase intention	0.530	0.370	0.610	0.700	0.658	0.654	***0.877***

N = 331. Figures in italicized bold denote the square root of the AVE of each study construct.

### 4.2. Hypotheses testing

**Figure 2:**
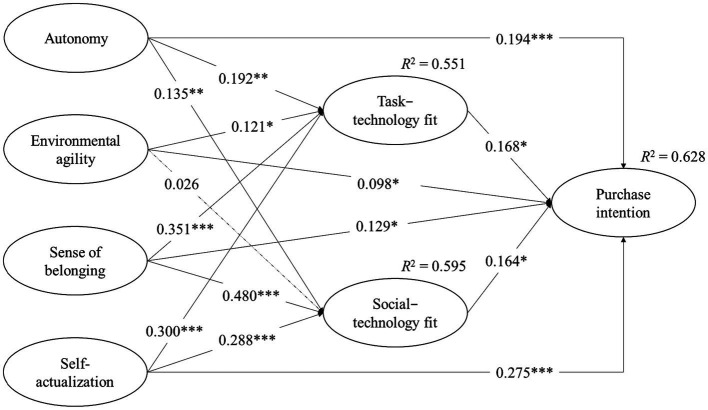
Results of the SEM estimations.

We tested our hypotheses empirically by performing SEM analyses after confirming the validity of the measures. Figure 2 presents the results of the SEM estimations. Hypotheses 1 and 2 predicted that autonomy and environment agility of GSHPs had a positive effect on task-technology fit, respectively. As shown in Figure 2, a positive and statistically positive relationship existed among autonomy (*b* = 0.192, *p* < 0.01), environmental agility (*b* = 0.121, *p* < 0.05), and task-technology fit. These results provided support for Hypotheses 1 and 2. Relatedly, Hypotheses 3 and 4 proposed that the autonomy and environment agility of GSHPs had a positive effect on social-technology fit. The results reported in Figure 2 indicated a positive and statistically significant relationship between autonomy (*b* = 0.135, *p* < 0.01) and social-technology fit, thus supporting Hypothesis 3. However, the results showed a positive but statistically insignificant effect of environmental agility on social-technology fit (*b* = 0.026, *p* > 0.05). Thus, Hypothesis 4 was not supported. Then, Hypotheses 5 and 6 proposed that the autonomy and environment agility of GSHPs had a positive effect on purchase intention. As shown in Figure 2, these variables, including the autonomy (*b* = 0.194, *p* < 0.001) and environment agility (*b* = 0.098, *p* < 0.05) of GSHPs, exerted positive and significant influences on purchase intention. Thus Hypotheses 5 and 6 were supported. We examined Hypotheses 7 and 8 by estimating the effects of a sense of belonging and self-actualization on task-technology fit. The results presented in Figure 2 showed a positive and statistically significant relationship among sense of belonging (*b* = 0.351, *p* < 0.001), self-actualization (*b* = 0.300, *p* < 0.001), and task-technology fit. These results demonstrated respective support for Hypotheses 7 and 8. In Hypotheses 9 and 10, we hypothesized a positive effect of a sense of belonging and self-actualization on social-technology fit. As shown in Figure 2, a positive and statistically significant relationship existed between a sense of belonging (*b* = 0.480, *p* < 0.001), self-actualization (*b* = 0.288, *p* < 0.001), and social-technology fit. These results provided support for Hypotheses 9 and 10, respectively. Furthermore, Hypotheses 11 and 12 proposed that a sense of belonging, and self-actualization had a positive effect on purchase intention, which could be verified by the results in Figure 2. Sense of belonging (*b* = 0.129, *p* < 0.05) and self-actualization (*b* = 0.275, *p* < 0.001) had positive and significant influences on purchase intention, thus providing support for Hypotheses 11 and 12. In addition, we empirically examined Hypotheses 13 and 14, which proposed the effects of task-technology fit and social-technology fit on purchase intention, respectively. The results reported in Figure 2 showed that task-technology fit (*b* = 0.168, *p* < 0.05) and social-technology fit (*b* = 0.164, *p* < 0.05) had positive and statistically significant effects on purchase intention, thus supporting Hypotheses 13 and 14.

Finally, while it goes beyond the scope of our study, we raised the question of whether an indirect effect is exerted via task-technology fit or social-technology fit. Accordingly, we examined the potential indirect effects to supplement our analysis and report the results for indirect effects testing in Table 3. As shown in Table 3, we found a positive and statistically significant indirect effect of autonomy on purchase intention at a 10% level via task-technology fit (*b* = 0.032, *p* < 0.10) and social-technology fit (*b* = 0.022, *p* < 0.10). This outcome suggested a partial mediating effect of task-technology fit and social-technology fit on the relationship between autonomy and purchase intention (i.e., both direct and indirect effects of autonomy on purchase intention were positive and significant). We also found that sense of belonging has a positive and significant indirect effect on purchase intention at a 5% level via both task-technology fit (*b* = 0.059, *p* < 0.05) and social-technology fit (*b* = 0.079, *p* < 0.05). Given its positive and significant direct effect on purchase intention, these results also indicated a partial mediating effect of both task-technology fit and social-technology fit on the relationship between a sense of belonging and purchase intention. Moreover, the results reported in Table 3 demonstrated a positive and significant indirect effect of self-actualization on purchase intention via task−technology fit (*b* = 0.050, *p* < 0.10) at the 10% level and social-technology fit (*b* = 0.047, *p* < 0.05) at the 5% level. Considering the positive and significant direct effect of self-actualization on purchase intention, these results demonstrated that task-technology fit and social-technology fit played an important role in partially mediating the effect of self-actualization on purchase intention. However, we found no significant indirect effect of environmental agility on purchase intention via either task-technology fit (*b* = 0.020, *p* > 0.10) or social-technology fit (*b* = 0.004, *p* > 0.10), thus providing no evidence of the mediating effect of either task-technology fit or social-technology fit in the relationship between environmental agility and purchase intention. We discuss the detailed results and their potential implications in the next section.

**Table 3:** Results of structural model estimation for indirect effects.

Indirect effects	Estimates	*p* values
Autonomy → Task-technology fit → purchase intention	0.032^*^	0.065
Autonomy → Social-technology fit → purchase intention	0.022^*^	0.080
Environmental agility → Task-technology fit → purchase intention	0.020	0.105
Environmental agility → Social-technology fit → purchase intention	0.004	0.603
Sense of belonging → Task-technology fit → purchase intention	0.059^**^	0.026
Sense of belonging → Social-technology fit → purchase intention	0.079^**^	0.032
Self-actualization → Task-technology fit → purchase intention	0.050^*^	0.058
Self-actualization → Social-technology fit → Purchase intention	0.047^**^	0.043

* *p* < 0. 10.

** *p* < 0. 05.

## 5. Discussion and conclusion

### 5.1. Discussion and implications for theory and practice

In this study, building upon the perspective of the hierarchy of needs and task-technology fit, we theorize and empirically explore how to promote the purchase intention of GSHPs by achieving a fit between the technology characteristics of GSHPs and consumers’ needs. In doing so, we developed a theoretical model to specifically analyze how the alignment between the technology characteristics of GSHPs and consumers’ needs influences consumption decision-making. We believe our study provides an important contribution to the literature by offering a fresh perspective on the application of the hierarchy of needs and task-technology fit theories. In particular, the integration of these two prominent theories in this study contributes to the literature on consumers’ purchase intentions for GSHPs by offering a comprehensive perspective on the factors influencing consumer behavior in the context of sustainable and technologically advanced home solutions. We believe such theoretical synthesis enhanced our insights into the complex dynamics of consumer behavior and would help pave the way for more effective strategies in promoting sustainable and environmentally friendly technologies. Overall, our study provides several implications which can be summarized as follows.

First, this study finds that the autonomy of GSHPs has a positive impact on both task-technology fit and social-technology fit. However, environmental agility only has a positive impact on task-technology fit. Previous research has found that the autonomy and environmental agility of GSHPs have the potential to enhance consumers’ user experience, improve interaction quality, and provide a platform for self-improvement (Cho and Kim, 2014). The current study extends these findings by demonstrating that the autonomy and environmental agility of GSHPs can improve task-technology fit. This result emphasizes the significance for manufacturing enterprises to improve the autonomy and environmental agility of GSHPs continuously. For example, the ability of smart objects must be enhanced to distinguish different dialects or accents to make accurate responses to users’ commands from different regions and age groups. Moreover, the level of intelligent automation and the accuracy of inbuilt sensors for environment monitoring must be improved.

However, environmental agility does not have a positive impact on social-technology fit. A possible explanation for this result is that, while the autonomy of GSHPs implies an instant response, which allows users to provide real-time feedback based on instructions, environmental agility may require intelligent devices to scan, perceive, learn, and adapt to the environment before reacting (Rijsdijk and Hultink, 2009; Rokonuzzaman et al., 2022). This delay of gratification may affect consumers’ attitudes toward smart home technology, and the satisfaction degree of social needs may be significantly reduced. Smart home manufacturers need to improve and innovate smart home technology continuously to enhance the capabilities of their products for information process and quick response to improve social-technology fit.

Second, the positive relationship between technology characteristics and consumers’ purchase intention has also been verified. Previous research has examined the mediating effects between autonomy and experience value, an antecedent of repurchase intention for smart products. However, it has denied the direct effect of autonomy on repurchase intention (Lucia-Palacios and Pérez-López, 2023). By contrast, this study provides statistical evidence for the direct effect and highlights the significance of technological enhancement.

Moreover, this study finds that consumers’ needs for a sense of belonging and self-actualization have positive effects on task-technology fit and social-technology fit, respectively. It implies the importance for firms to pay attention to consumers’ needs for belongingness and self-actualization when considering the fit among technology, tasks, and social demands. Firms need to connect additional devices and users to their intelligent platforms to increase the quantity and quality of interactions. Sense of belonging can be enhanced through interaction with their smart devices within their houses and through sharing information about the smart home devices with their relatives and friends (Lee et al., 2017).

More importantly, when consumers want to establish a better self-image or demonstrate a higher social status, they are likely to select unique products with high technology characteristics and engage in conspicuous consumption to satisfy their social needs (Gram-Hanssen and Darby, 2018). Some of these consumers may even sacrifice some benefits in price to satisfy their pursuit of a higher social status (Ramakrishnan et al., 2020). Inspired by this result, smart home enterprises can adopt different strategies for consumers with different levels of task needs and social needs. Marketers can also enhance buyers’ green and environmentally friendly image through the promotion of the environmental performance of GSHPs so that more consumers may be attracted to increase their psychological needs by purchasing GSHPs.

In addition, the results also indicate a positive effect of a sense of belonging and self-actualization on consumers’ purchase intention, which is highly in line with the previous findings (Hwang and Kim, 2018; Hawkins, 2020; Lebrument et al., 2021). Thus, the current study highlights the importance of satisfying consumers’ needs.

Finally, this study confirms that task-technology fit and social-technology fit have a positive effect on consumers’ purchase intentions for GSHPs, as emphasized by previous research (Lu and Yang, 2014; Ling et al., 2021). This study extends these findings by showing that task-technology fit and social-technology fit should be considered when firms promote consumers’ purchase intentions for GSHPs. In the production and marketing process, enterprises should not only focus on the technology characteristics of GSHPs and the features of consumers’ needs but also analyze the degree of fit among technology characteristics, tasks, and social needs of their consumers. A higher degree of fit can provide consumers with an optimal consumption experience, increase their purchase intention, and help enterprises improve their competitiveness and performance.

### 5.2. Limitations and future research directions

This study has certain limitations due to several factors, such as time, energy, and region. First, this study selected Chinese consumers as the research sample. However, the differences in technology level and consumers’ demand for GSHPs in different countries may affect the universality of the research results. Further research can expand the research scope by studying consumers’ purchase intention of GSHPs in different regions with various technological, economic, and cultural backgrounds (*cf.* Li et al., 2021). Second, previous studies have found that factors such as consumers’ gender, social status, and education level may affect their acceptance of innovative technology products (Venkatesh et al., 2000; Cai et al., 2017; Shin et al., 2018). Therefore, future research can attempt to explore the impact of these factors on consumers’ purchase behavior of GSHPs based on this study. Third, this study verifies the positive impact of the autonomy and environmental agility of GSHPs on purchase intention. Scholars have also identified other characteristics of smart products, such as anthropomorphism, cooperativeness, and connectivity (Rijsdijk and Hultink, 2009; Langley et al., 2021). Subsequent research can expand the research scope of the technology characteristics of GSHPs to obtain a comprehensive analysis of green purchase behavior for smart products.

## Data availability statement

The raw data supporting the conclusions of this article will be made available by the authors, without undue reservation.

## Ethics statement

Ethical approval was not required for the studies involving humans in accordance with the national legislation and the institutional requirements. The studies were conducted in accordance with the local legislation and institutional requirements. The participants provided their written informed consent to participate in this study.

## Author contributions

MG: Visualization, Conceptualization, Data curation, Investigation, Writing – original draft. SX: Conceptualization, Visualization, Formal analysis, Methodology, Supervision, Validation, Writing – review & editing.
